# Facile Modification of NF Membrane by Multi-Layer Deposition of Polyelectrolytes for Enhanced Fouling Resistance

**DOI:** 10.3390/polym13213728

**Published:** 2021-10-28

**Authors:** Umair Baig, Abdul Waheed, Hassan A. Salih, Asif Matin, Ali Alshami, Isam H. Aljundi

**Affiliations:** 1 Interdisciplinary Research Center for Membranes and Water Security, King Fahd University of Petroleum and Minerals, Dhahran 31261, Saudi Arabia; umairbaig@kfpum.edu.sa (U.B.); Abdulwaheed@kfupm.edu.sa (A.W.); amatin@kfupm.edu.sa (A.M.); 2 College of Engineering, Khalifa University, Abu Dhabi 127788, United Arab Emirates; Hassan.hussein@ku.ac.ae; 3 Chemical Engineering Department, University of North Dakota, Grand Forks, ND 58202, USA; ali.alshami@und.edu; 4 Chemical Engineering Department, King Fahd University of Petroleum and Minerals, Dhahran 31261, Saudi Arabia

**Keywords:** polyelectrolytes, poly(allylamine hydrochloride), poly(acrylic acid), membrane fouling, nanofiltration, surface modification

## Abstract

Fouling not only deteriorates the membrane structure but also compromises the quality of the permeate and has deleterious consequences on the membrane operation. In the current study, a commercial thin film composite nanofiltration membrane (NF90) was modified by sequentially depositing oppositely charged polycation (poly(allylamine hydrochloride)) and polyanion (poly(acrylic acid)) polyelectrolytes using the layer-by-layer assembly method. The water contact angle was decreased by ~10° after the coating process, indicating increased hydrophilicity. The surface roughness of the prepared membranes decreased from 380 nm (M-0) to 306 nm (M-10) and 366 nm (M-20). M-10 membrane showed the highest permeate flux of 120 L m^−2^ h^−1^ with a salt rejection of >98% for MgSO_4_ and NaCl. The fabricated membranes M-20 and M-30 showed 15% improvement in fouling resistance and maintained the initial permeate flux longer than the pristine membrane.

## 1. Introduction

A continuous supply of clean and potable water is not only crucial for the survival of living organisms but also equally important for perpetual industrial processes. Membrane-based separations are gaining increased attention from the research community due to unique features such as modularity and low specific energy requirements [1,2]. Based on the average pore size, pressure-driven membrane processes are classified into four categories, namely microfiltration (MF) with pore size of 0.1 µm to 5.0 µm, ultrafiltration (UF) with pores of 20 nm to 0.1 µm, nanofiltration (NF) with pores of >1 nm, and reverse osmosis (RO) with pores of the order of 0.1 nm to 1.0 nm [3]. Among other types of membranes, thin film composite (TFC) polyamide (PA) membranes have played a significant role as NF and RO membranes. The NF membranes are playing their part by maintaining the high quality of a water supply, softening the hard water and treating wastewater along with applications in food industry [4].

Despite the tremendous potential of polyamide TFC-NF membranes for water purification, such membranes suffer from a serious challenge of progressive fouling by organic and biofoulants present in the feed. Membrane fouling not only affects the quantity and quality of the permeate but also leads to membrane deterioration and decrease in the life span of the membrane which in turn can potentially lead to increased capital cost. Furthermore, the irreversible deposition of foulants on the membrane surface results in reduced performance of the membrane which necessitates an increase in the applied transmembrane pressure, thus creating further increase in operational costs [5]. Hence, the surface engineering of polyamide TFC-NF membranes is desperately needed to address the hostile challenges associated with membrane fouling.

Surface modification of membranes has attracted the attention of researchers worldwide as a simple yet effective method for fouling control. The surface of polyamide TFC-NF membranes has been made more hydrophilic for the sake of mitigating the organic and biofouling of the polyamide membranes. It has been well established in the literature that the hydrophilic membrane surface leads to the formation of a strong hydration layer of water molecules through hydrogen bonding, and this hydration layer develops repulsive interactions with the foulants, which leads to lower fouling. Various strategies have been adopted in this regard and include surface grafting [6], coating [7], UV irradiation [8,9], plasma treatment [10], and nanomaterial incorporation [11]. A recent trend of incorporating hydrophilic nanomaterials has also emerged with great potential to hamper the growth of the foulants on the membrane. It has been observed that the nanoparticles with globular 3D structure not only enhance the antifouling performance of the membrane but also introduce additional water channels that keep the permeability of the membrane intact even after surface modification. Although such hydrophilic nanoparticles possess huge potential for enhancing the antifouling performance of the membrane, the incorporation of these nanoparticles suffers from serious drawbacks, such as the well-known agglomeration of nanoparticles during membrane fabrication, high cost, and tedious synthesis of nanoparticles for large-scale applications [12].

Another approach for making the membrane surface more hydrophilic is the coating of the surface with polyelectrolytes for tuning the membranes both physically and chemically [13]. The polyelectrolytes are deposited on the membrane surface using layer-by-layer (LBL) coating [14]. These coatings can be applied by spray-coating, spin-coating, and dip-coating [15]. In a recent study, Hu et al. synthesized a new antifouling TFC-NF membrane by incorporating hyperbranched polyglycerol (hPG) using the layer-by-layer interfacial polymerization. The hPG decorated membrane showed increased hydrophilicity with the contact angle reaching to 16.4° and inhibiting the membrane fouling by bovine serum albumin (BSA) [16]. In another work carried out by Mohtada et al., a combination of polyelectrolytes including poly(diallyldimethylammonium chloride) and poly(acrylic acid) (PAA) was used to modify a polyamide-imide ultrafiltration membrane. The effect of the number of bilayers was studied to find out the best conditions for an exceptionally performing antifouling membrane. The effective number of bilayers was found to be four, where the fabricated membrane showed hydrodynamic permeability of 5.2 (L m^−2^ h^−1^)/psi with a flux decline of 50.2% and flux recovery of 100%, while the flux decline of the pristine membrane was 75.9% with a flux recovery of 97.8% [17]. Therefore, the selection of an appropriate polymeric cationic–anionic pair is a key for LBL technique leading to the fabrication of a promising antifouling membrane.

Herein, NF90 polyamide TFC-NF membrane was modified through LBL coating technique. Poly(allylamine hydrochloride) and poly(acrylic acid) were used as polycation and polyanion, respectively. The pristine and modified membranes were thoroughly characterized to confirm their surface and structural characteristics. The performance of the membranes was investigated in terms of permeate flux and salt rejection using a customized crossflow laboratory setup. The antifouling performance of the membranes was studied by using a synthetic fouling solution of humic acid, and the permeate flux was monitored over time.

## 2. Materials and Methods

### 2.1. Materials

**Chart 1:**
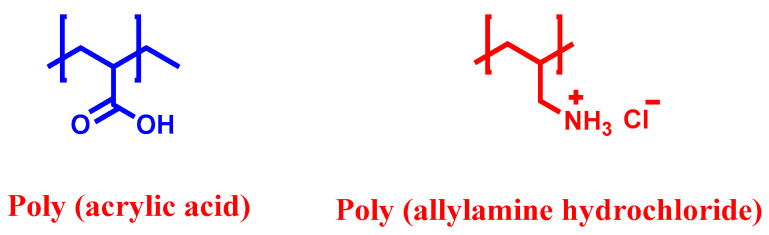
The chemical structures of PAA and PAH.

Isopropanol (>99.7%), poly(allylamine hydrochloride) (PAH, Mw 120,000–180,000 g/mol), poly(acrylic acid) (PAA, Mw 25,000–250,000 g/mol), sodium chloride (NaCl, >99.5%), magnesium sulfate (MgSO_4_, >99.5%), and humic acid were purchased from Sigma-Aldrich and used without further treatment. NF90 commercial polyamide NF membrane (Sterlitech, Auburn, WA, USA) was also used. The structures of the poly(acrylic acid) and poly(allyl amine hydrochloride) are given below in Chart 1.

### 2.2. Preparation of the Polyelectrolyte LBL Assembled Membranes

The nanofiltration membrane was synthesized by dip-coating and layer-by-layer deposition of polyelectrolyte precursors, namely PAA and PAH. The commercial polyamide (PA) membrane (NF90) was soaked in isopropanol for approximately 45 min and was thoroughly rinsed by deionized (DI) water afterwards. The PA membrane was then immersed in an aqueous solution of PAH (1000 ppm) for 1 min and rinsed with DI water and was subsequently dipped in an aqueous solution of PAA (1000 ppm) for one minute followed by rinsing with DI water. This procedure was repeated to achieve the required number of layers such as 10, 20 and 30 layers. The modified membranes were named according to the number of deposited layers: M-0 (pristine polyamide membrane), M-10, M-20 and M-30. The prepared membranes were then dried at ambient conditions before filtration experiments.

### 2.3. Membrane Characterization

The presence of various functional ties in the pristine and the modified membranes was investigated by attenuated total reflectance Fourier transform infrared spectroscopy (ATR-FTIR; Nicolet 6700, Thermo Scientific, Waltham, MA, USA). A perfectly dried membrane sample was held in the ATR mode and the FTIR spectra were recorded over the range of 400–4000 cm^−1^. The surface morphologies of the pristine and modified membranes were analyzed using a TESCAN field emission scanning electron microscope (FE-SEM, TESCAN USA, Inc., Warrendale, PA, USA) at 10 kV. Prior to FESEM analysis, the membrane samples were coated with 5 nm gold layer using an ion sputter coater (Q150R, Quorum Technologies, Lewes, UK). The surface roughness of the prepared membranes was studied by using a VEECO Dimension 3100 for atomic force microscopy (AFM, Veeco Instruments Inc, Santa Barbara, CA, USA). The surface hydrophobicity and wettability features were studied by measuring the water contact angle (WCA) using the sessile drop method (DSA25, Kruss GmbH, Hamburg, Germany). A 2 µL drop was deposited onto the surface of the dry membrane sample and the contact angle was measured after the equilibrium was reached. SDT Q600 TGA (TA Instruments, New Castle, DE, USA) was used for the thermogravimetrical analysis (TGA) in a temperature range of 30–800 °C at a heating rate of 10 °C/min under a nitrogen flow of 100 mL/min.

### 2.4. Nanofiltration Performance of the Membranes

**Figure 1:**
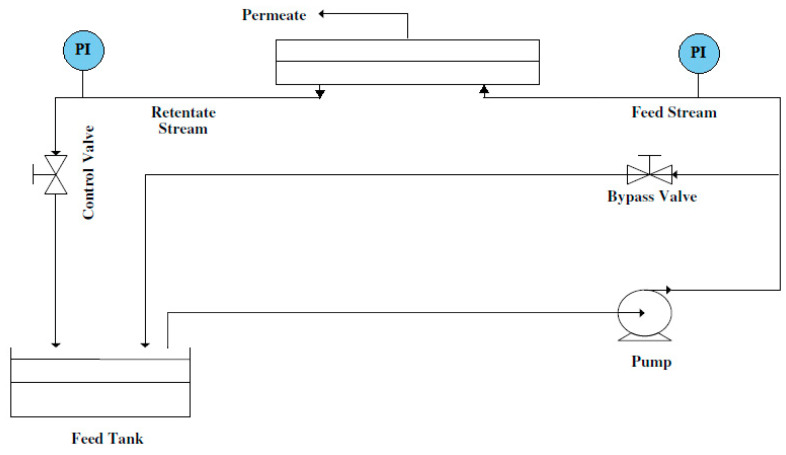
Schematic sketch of the lab-scale experimental setup used for the membrane filtration studies.

A laboratory-scale crossflow filtration setup was used for evaluating the performance of the membranes (Figure 1). This setup consisted of a rectangular-shaped membrane cell (CF016, Sterlitech, Auburn, WA, USA) with an active area of 20.6 cm^2^, a high-pressure pump (Wanner Engineering, Inc., Minneapolis, MN, USA) for providing hydraulic transmembrane pressure (TMP) and a feed tank inter-connected with Teflon and stainless steel tubing. A stand-alone recirculating chiller (Cole Parmer, Inc., Vernon Hills, IL, USA) was used to control the feed water temperature. Prior to the nanofiltration experiments, the membranes were compacted at 20 bar until a steady permeate flux was obtained. Salt retention experiments were performed at 1000 ppm NaCl or MgSO_4_, at TMP of 20 bar, and a temperature of 23 ± 1 °C.

The permeate flux was calculated using the following formula:

(1) $$J=\frac{V}{A\times t}$$

where *J* is the permeate water flux in liters per square meter per hour (LMH), *V* (L) is the volume of permeate collected during time, *t* (h), and *A* (m^2^) is the membrane area. A conductivity meter (HI9813-5N, Hanna Instruments, Woonsocket, RI, USA) was used to measure the conductivities of the feed and the permeate waters to quantify the percent salt rejection.

The permeate flux can be expressed in terms of the driving force as:

(2) J=A(∆P−∆π)

where A is the effective membrane permeance to water, and ∆P is the transmembrane pressure, while ∆π is the osmotic pressure difference across the membrane following the phenomenological Darcy’s law. When the permeate water has a negligible salt concentration, the permeate side’s osmotic pressure can be neglected. The osmotic pressure (π) of a solution can be calculated via the van ’t Hoff equation as:

(3) π=iCRT

where *i* is the van ’t Hoff factor (*i* = 2 for NaCl and MgSO_4_), *C* is the salt molar concentration (mol/L), *R* is the universal gas constant (8.314 J/mol/K), and *T* is the absolute temperature (K).

The apparent NaCl rejection was calculated with the following relation:

(4) $$R\;=\;\left(1\;-\;\frac{C_{p}}{C_{f}}\right)\;\times \;100$$

where *R* is the apparent salt rejection (%), and *Cp* and *Cf* are the concentration of the salts in the permeate and feed streams, respectively.

### 2.5. Evaluation of Antifouling Performance of the Membranes

Humic acid solution with a concentration of 100 ppm was used for the fouling experiments. In a typical experiment, the membrane was compacted using distilled water at a pressure of 20 bar and a temperature of 23 ± 1 °C until steady flux was reached. Afterwards, the distilled water was replaced with the humic acid solution and run at the same conditions of pressure and temperature while monitoring the permeate flux with time. The relative flux (RF) was calculated as the ratio $J/J_{0}$_._ where J is the permeate flux at time *t* and $J_{0}$ is the initial permeate flux at t=0.

## 3. Results and Discussion

### 3.1. Membrane Assembly and the Conceptual Model

**Figure 2:**
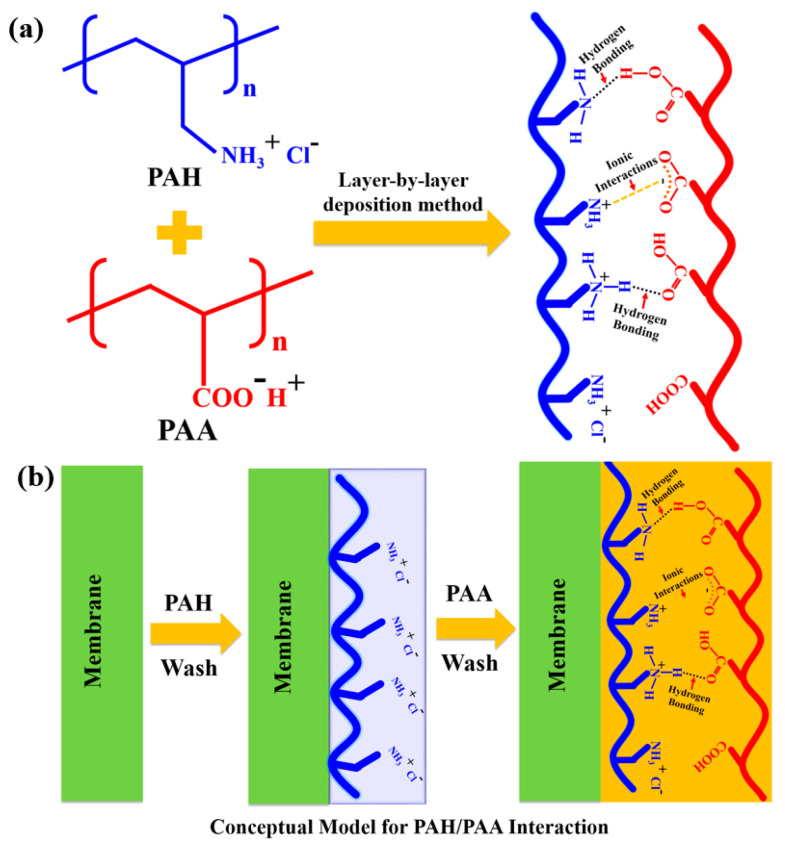
(**a**) Conceptual model for possible interactions of the PAA and PAH before and (**b**) LBL deposition on NF90 membrane.

The pristine polyamide (M-0) membrane was modified by sequential deposition of polycations and polyanions on the membrane surface. The polyelectrolytes are deposited onto the membrane by strong columbic interactions and hydrogen bonding. It is known that polyamide membranes have inherent negative charge, which is attributed to hydrolysis of residual acid chloride groups on the membrane surface. The LBL deposition utilizes the inherent negative charge of the NF90 membrane, leading to the deposition of the desired number of layers on the membrane [18]. The negatively charged M-0 was dipped in the PAH solution leading to the deposition of the polycations on the membrane rendering the surface of the membrane positively charged. The positively charged membrane readily interacts with the new incoming layer of PAA and hence a polycation–polyanion pair is formed on the membrane surface. The possible interactions of the PAA and PAH are given below in Figure 2. The -COOH group of PAA is both a hydrogen bond donor and acceptor with the possibility of tautomerism as the protons of the -COOH can easily exchange with other groups such as -NH_2_ leading to a delocalized negative charge on the -COO- function of the PAA that develops strong electrostatic interaction with the positively charged ammonium ions of PAH.

### 3.2. The Surface Functionalities

**Figure 3:**
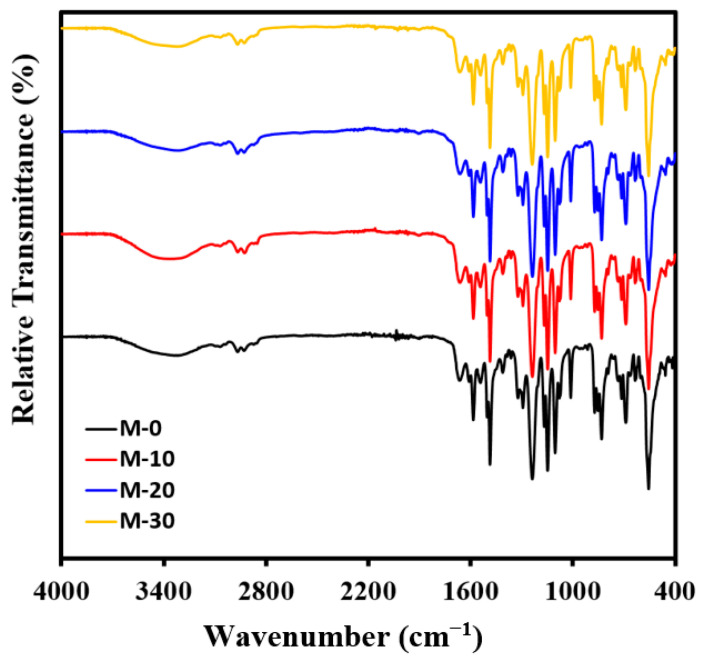
Complete FTIR Spectra of M-0, M-10, M-20 and M-30 membranes.

The complete FTIR spectra of the pristine and the modified membranes are given in Figure 3. No remarkable variation was observed in the FTIR spectra of the prepared membranes indicating that the functionalities present in the pristine and modified membranes are the same. The broad peak present in the range of 3600 cm^−1^ to 3200 cm^−1^ was attributed to the presence of amide linkage (-CO-NH) overlapped by -COOH functions present in the membranes, while the peaks located at 2900 cm^−1^ and 2800 cm^−1^ were due to aliphatic -CH_2_ and -CH bonds. Moreover, the presence of carbonyl function (>C=O) was confirmed at 1650 cm^−1^ [16,17]. Similarly, the fingerprint regions of all of the membranes resembled each other, which further hints that the deposited layers were quite thin since the characteristic peaks of the M-0 membrane were significantly visible even after the LBL modification.

### 3.3. Morphological Features of the Membranes

**Figure 4:**
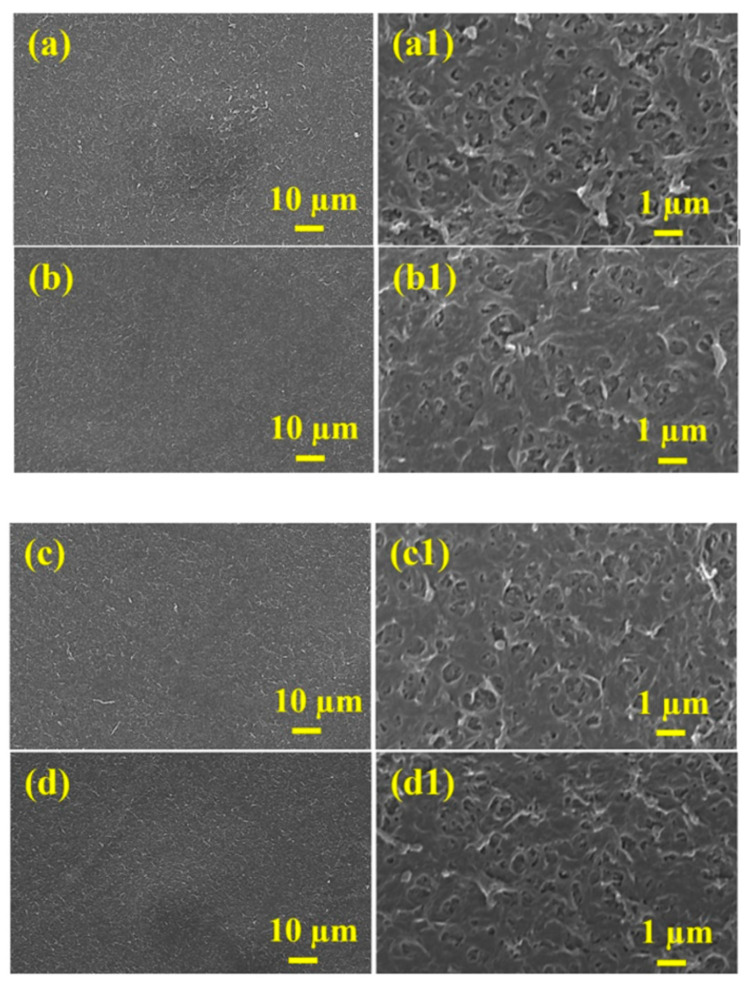
FESEM micrographs of (**a**,**a1**) pristine M-0, (**b**,**b1**) M-10, (**c**,**c1**) M-20 and (**d**,**d1**) M-30.

The surface morphological features of the pristine and modified membranes were thoroughly investigated by FESEM analysis as illustrated in Figure 4. It is clearly evident from the surface micrographs of the pristine membrane (M-0) that it has a crinkled and rough surface with irregularities and microvoids. This ridge-and-valley structure is a common feature of the commercial polyamide membranes that is attributed to the successful interfacial polymerization between the reacting monomers. However, after the deposition of the polyelectrolyte pairs on the pristine membrane, the surface morphological features of M-0 were slightly altered as the surface became progressively smoother than the pristine membrane, which dictated the success of the LBL surface deposition of polyelectrolytes on M-0. A relatively smooth membrane is also ideal for reducing the membrane surface fouling. The effect of surface modification increased with the increasing number of deposited layers as evidenced by the FESEM micrographs shown in Figure 4. This result is consistent with previous studies found in the literature [19,20].

### 3.4. Surface Roughness and Wettability

**Figure 5:**
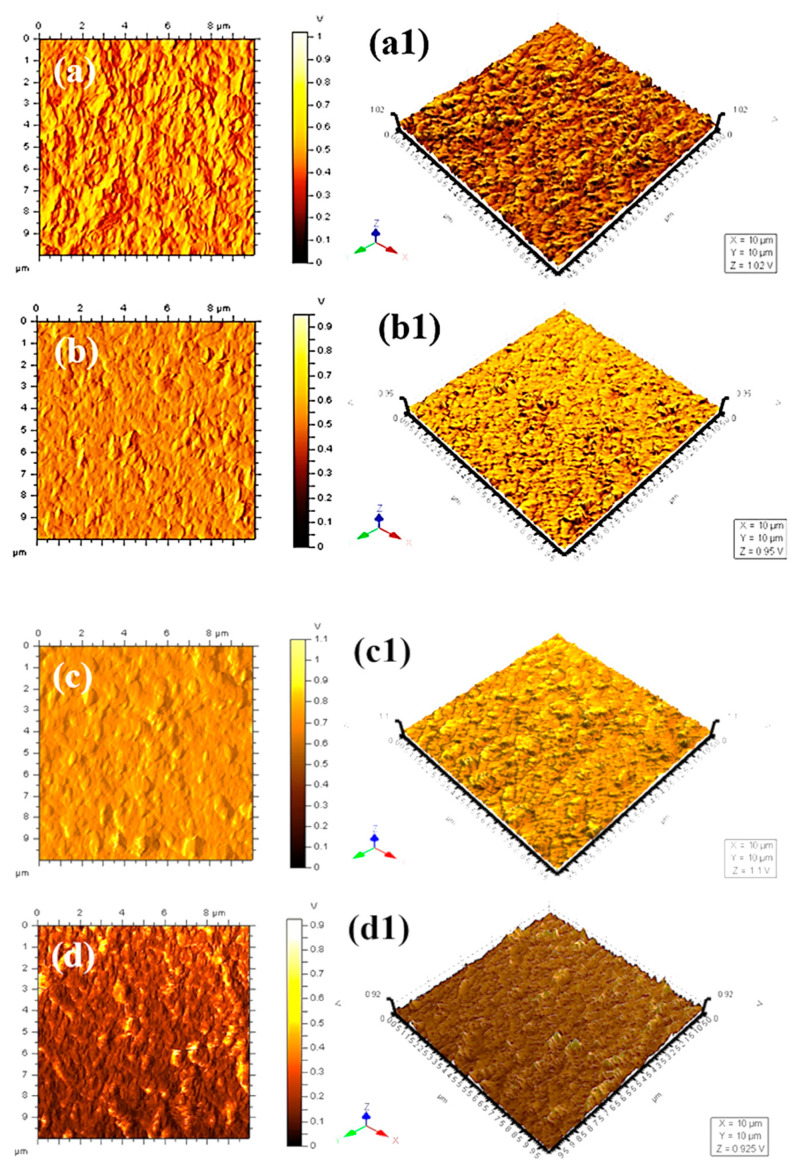
Three-dimensional AFM images for the membranes (**a**,**a1**) M-0, (**b**,**b1**) M-10, (**c**,**c1**) M-20, and (**d**,**d1**) M-30.

**Figure 6:**
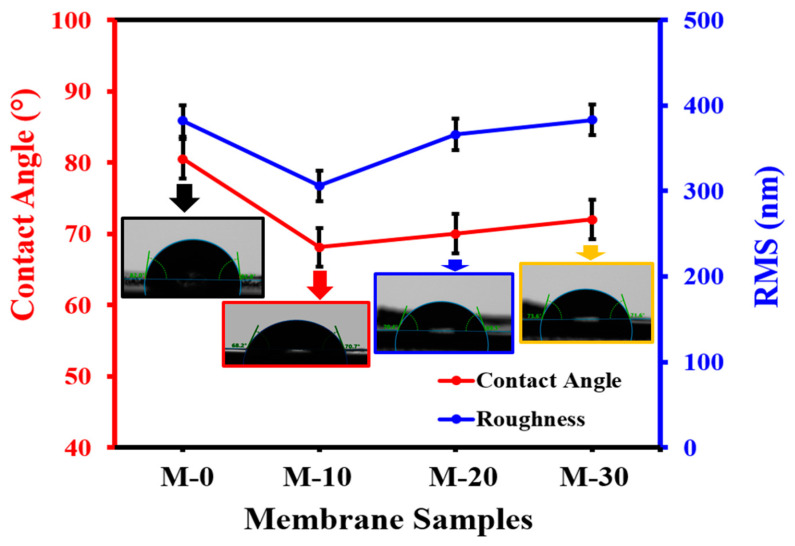
Water contact angle and root mean square roughness for the prepared membranes.

The surface roughness of the membranes is crucial and critically important during the evaluation of the antifouling performance of the membranes. Figure 5 displays the AFM images for the membranes before and after surface modification. The images supported the expected enhancement by LbL modification, where surface grooves and irregularities were filled by polyelectrolytes [21,22]. Initially, the root mean square (RMS) roughness decreased with the deposition of polyelectrolyte layers (Figure 6). Generally, it is believed that the decreased surface roughness of the polyamide membrane is highly desirable for enhancing antifouling performance of the membranes. The M-10 and M-20 showed the smooth surface after the deposition of polyelectrolytes. However, an increase in the membrane roughness was observed for M-30, which might be attributed to the deposition of large number of polyelectrolyte layers. Hence, it is strongly recommended to exert great caution when applying the LBL deposition strategy for membrane modification [23,24].

Similarly, the hydrophilicity of the membrane significantly affects its intrinsic permeability and antifouling properties. The water contact angle (WCA) is used to determine the membrane hydrophilicity and interfacial tension between water and membrane surface. The WCAs of all membranes are given in Figure 6. The WCA showed a significant decrease from 82.0° of M-0 to 68.2°, 70.4° and 73.6° for M-10, M-20 and M-30, respectively. The decrease in WCA is attributed to the hydrophilicity of the polyelectrolytes as the ammonium ions (-NH_3_^+^) of PAH and carboxylic group (-COOH) of PAA have strong affinity for water. Both -NH_3_^+^ and -COOH groups can develop hydrogen bonding with the water molecule, which can in turn help in the formation of a strong hydration layer on the modified membranes which can in turn reduce the membrane fouling [25]. Hence, the deposition of polyelectrolyte layers on the TFC membranes can potentially be an important strategy for making the membrane more hydrophilic.

### 3.5. Thermogravimetric Analysis of the Membranes

**Figure 7:**
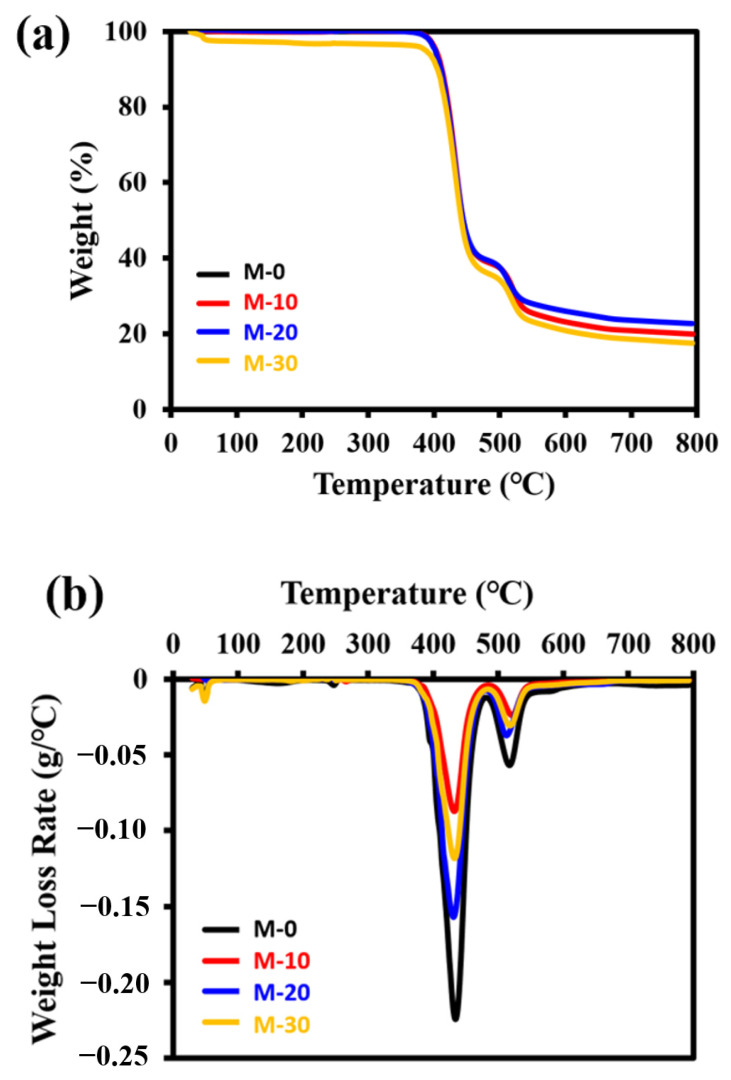
(**a**) The TGA profile and (**b**) the DTG analysis of the prepared membranes.

The thermal stability of the pristine and the modified membranes was studied by thermogravimetric analysis (TGA). All membranes exhibited a similar weight loss pattern (Figure 7) and were found to be highly stable up to 400 °C, while a sharp loss of mass (60%) was observed with a decomposition temperature of 430 °C. However, M-30 showed a slightly higher mass loss which might be attributed to the deposition of 30 additional layers on M-0 (Figure 7a). Moreover, the DTG analysis (Figure 7b) was also carried out to determine the exact temperature and the steps involved during the thermal degradation of the membranes. The DTG analysis indicated that the membranes were degraded in two major steps located at 430 °C and 530 °C. The first step at 430 °C can be attributed to the thermolysis of the polyamide and polyelectrolytes chains with 60% loss of mass. The second step corresponds to 20% loss of mass, which might be attributed to thermo-oxidative degradation and carbonization of the polymer chains of the membranes leading to a total 80% weight loss (Figure 7b). The initial loss of mass can be attributed to the evaporation of the adsorbed water by the membrane [26].

### 3.6. Evaluation of Membrane Performance

**Figure 8:**
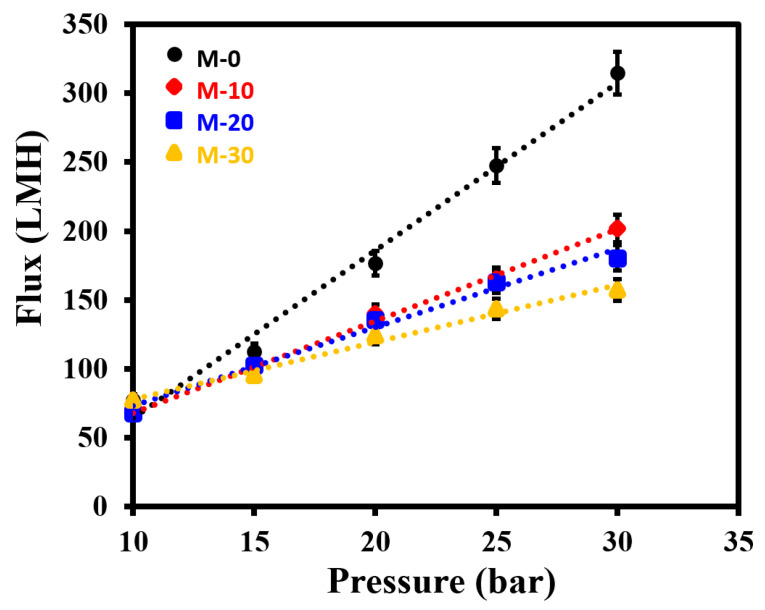
Pure water flux as a function of applied pressure for the original and modified membranes.

The pure water flux of all membranes was calculated and plotted against pressure as illustrated in Figure 8. The deposition of polyelectrolyte layers resulted into a slight decrease in pure water flux which can be explained by the increased resistance for mass transfer due to the extra polyelectrolyte layers [25,26]. However, as the pressure increased, the permeate flux increased linearly according to Equation (2), showing similar patterns for all membranes. The flux gap grew between the parent M-0 membrane and the LBL modified membranes to reach a maximum at 30 bar. At any pressure, the highest water flux was observed for the pristine M-0 membrane while other membranes showed a decreasing trend with the number of layers. Although the deposition of the multiple polyelectrolyte layers increased the antifouling performance of the membranes, the modified membranes suffered from a slight decrease in permeate flux [27,28].

**Figure 9:**
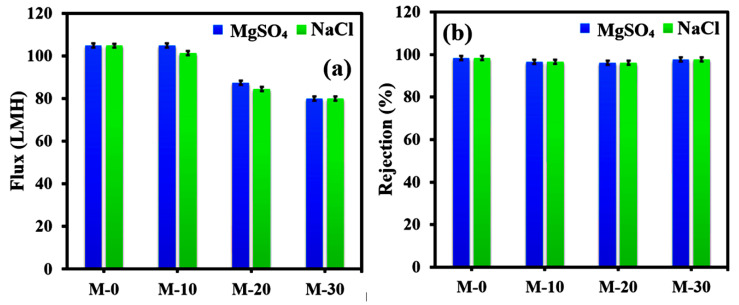
(**a**) Permeate flux and (**b**) salt rejection of all membranes at 20 bar, 25 °C, and 1 g/L salt.

The prepared membranes were tested for NaCl and MgSO_4_ rejection at 20 bar. The highest water flux was observed for the pristine membrane (120 LMH) while M-10 and M-20 and M-30 displayed 100, 84.5, and 80 LMH, respectively (Figure 9a). This drop in the permeate flux was associated with the drop in the effective membrane permeance (A) of M-0 from 12.18 LMH/bar to 6.68, 5.71, 4.14 LMH/bar for M-10, M-20, and M-30, respectively. The decrease in the permeate flux with the increase in the number of polyelectrolyte layers can be explained by the increased resistance for mass transfer. As presented in Figure 9b, the rejection of NaCl and MgSO_4_ reduced slightly with the increase of polyelectrolyte layers coated over the pristine membrane. This drop in rejection can be justified by the change in surface chemistry, where the growth of the charge density provided mobility for Na^+^ and Mg^2+^ ions by ion exchange [29,30]. Given the larger ionic radius of the divalent ions, the rejection of divalent Mg^2+^ cations were slightly higher than monovalent cations such as Na^+^ [31]. All of this may suggest that electrostatic repulsion and size exclusion mechanisms played a significant role in salt rejection.

**Figure 10:**
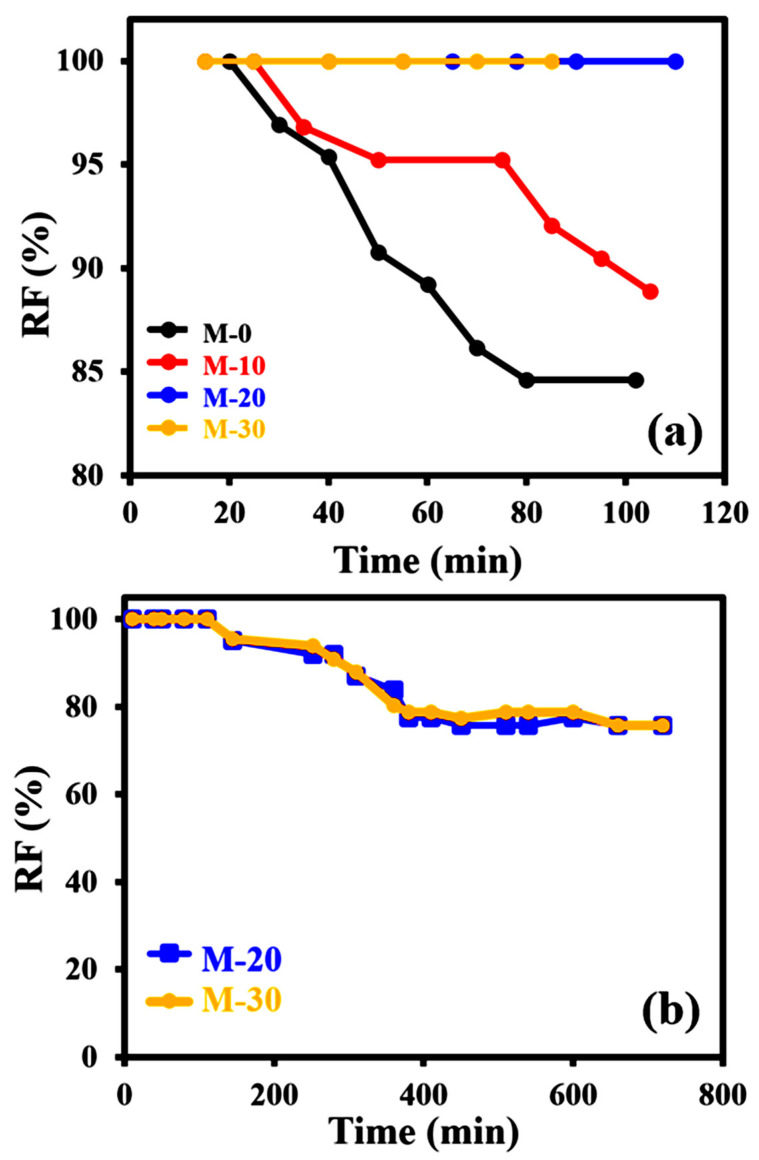
(**a**) Relative flux of all membranes after fouling by humic acid and (**b**) stability of M-20 and M-30 over 12 h.

The fouling behavior of all membranes was examined by feeding humic acid solution of 100 ppm to the membranes at a pressure of 20 bar. The M-0 membrane had a high fouling rate, as shown in Figure 10a, where it lost approximately 15% of its flux after 80 min. The flux stability of the membrane was slightly increased when coated with 10 layers of polyelectrolyte. Deposition of 20 and 30 layers boosted the flux stability compared to the parent M-0 membrane, which maintained the same initial flux for more than 90 min. The positive effect of the polyelectrolyte layers is attributed to the roughness reduction, charge density, and hydrophilicity, as confirmed by FESEM, AFM, and water contact angle. The foulant species had lower adhesion propensity to the modified membrane surface due to a smooth, charged, and hydrophilic surface. Although LBL assembly causes a slight increase in overall membrane thickness, the antifouling performance of modified membranes was significantly increased.

Given the good performance of the M-20 and M-30, these two membranes were selected for the long-term stability test over 12 h of continuous permeation with a fouling precursor solution, humic acid, at 20 bar. The permeate flux was maintained for approximately initial two hours of operation, followed by depreciation due to an accumulation of the foulant species over the membrane surface as shown in Figure 10b. This flux stability confirms the enhancement of surface characteristics such as improved charge density and hydrophilicity. Both M-20 and M-30 membranes exhibited less than 25% loss in permeate flow due to fouling after 700 min of continuous operation. However, the pristine M-0 showed a decline of 20% in permeate flow in just 100 min of continuous operation (Figure 10a) as M-0 is devoid of the features of improved hydrophilicity and charge density. This comparison reflects the promotional effect of polyelectrolyte deposition over NF membranes to reduce cost and energy required for operation.

Given the improved performance of the modified membranes such a strategy of depositing pairs of polyelectrolytes on TFC membranes can be utilized on a large scale to enhance the performance of the commercial polyamide membranes. As shown in Table 1, the features of the modified membrane are compared to similar membranes reported in literature.

## 4. Conclusions

This study demonstrates the successful modification of a commercial nanofiltration membrane using facile LBL assembly of polycation (PAH) and polyanion (PAA) polyelectrolytes. The modified membranes were found to be thermally stable and witnessed a decrease in surface roughness manifested by a decrease in RMS. Fouling studies with humic acid demonstrated that membranes coated with 20 and 30 bilayers of the polyelectrolytes had a significant fouling resistance and maintained the initial permeate flux for over 90 min while the flux of the pristine membrane declined by 15%. This combination of polyelectrolytes (PAA/PAH) enhanced fouling resistance of the NF membranes and has promising implications for future use.

## Figures and Tables

**Table 1:** Comparison of PAA–PAH modified polyamide NF membrane with other NF membranes.

Membrane	Water Contact Angle (°)	Membrane Testing Conditions	Flux (LMH)	Salts Rejection (%)	Antifouling Performance	Reference
TFN-PEI-GO-20 NF membrane	~35	2000 ppm Na_2_SO_4_; 5 bar; 25 °C	70.3	91%	Better than pristine membrane	[32]
TFN-GHC-60 NF membrane	~27	2000 ppm Na_2_SO_4_; 6 bar; 25 °C	48.0	>95%	Better than pristine membrane	[33]
Organic nanobowl containing TFN NF Membrane	~55	1000 ppm Na_2_SO_4_; 6 bar; 25 °C	100	~85%	Better than pristine membrane	[34]
Polyamide NF membranes modified with PVA	-	3000 ppm MgSO_4_; 6 bar; 25 °C	45.9	95.9	Improved by adding PVA	[35]
Polyamide NF membranes modified with chitosan	-	1000 ppm Na_2_SO_4_; 3 bar; 25 °C	59.6	98%	Improved by adding chitosan	[36]
PAA–PAH NF membrane	~70°	1000 ppm MgSO_4_; 20 bar; 25 °C	120	>98%	Excellent fouling resistant by adding PAA–PAH	This Study

## Data Availability

Data is contained within the article.
